# Data-driven identification of co-morbidities associated with rheumatoid arthritis in a large US health plan claims database

**DOI:** 10.1186/1471-2474-11-247

**Published:** 2010-10-25

**Authors:** Hans Petri, Debra Maldonato, Noah Jamie Robinson

**Affiliations:** 1 Epidemiology and Patient Reported Outcomes, Roche Products Limited, Shire Park, 6 Falcon Way, Welwyn Garden City AL7 1TW, UK; 2 Epidemiology and Patient Reported Outcomes, Roche Products, 340 Kingsland Street, Nutley, NJ 07110, USA; 3 Epidemiology and Patient Reported Outcomes, Hoffmann-La Roche, Building 663, Grenzacherstrasse 124, 4070 Basel, Switzerland

## Abstract

**Background:**

In drug development, it is important to have an understanding of the full spectrum of co-morbidities to be expected in the group of patients with the disease of interest. It is usually a challenge to identify the less common events associated with the target disease, even if these events are severe. The purpose of this study is to identify co-morbidities associated with rheumatoid arthritis (RA) as compared with a control group, using a large health care database.

**Methods:**

Marketscan US claims database was used for this retrospective cohort study. Selected were records of persons aged at least 16 Y with at least two claims for RA, and with active insurance status on June 30,2007. The control group had at least two claims for eczema/dermatitis.

Controls were matched by age, gender and insurance status (Medicare or not). All co-morbidities with an ICD9 diagnostic code were identified in the RA and control groups, during a one-year window. Relative risks (RRs) were calculated. Diagnoses were rank-ordered by magnitude of RR. Codes covering RA and arthropathy were excluded. In order to get stable estimates, rank-ordering was performed for diagnoses occurring in at least 20 persons in the control group.

**Results:**

Records were selected of 62,681 persons with RA (mean age was 59.0 Y, with 73.8% female, Medicare-covered 35%). A total of 6897 different ICD9 diagnostic codes were recorded, with 2220 codes in at least 20 persons of the control group [listed with Relative Risk]. Apart from joint/bone related conditions, strong associations with RA (RR > 3) were found for Adverse effect medicinal and biological substance not elsewhere classified, Unspecified adverse effect drug properly administered, Idiopathic fibrosing alveolitis, Osteomyelitis, Immune deficiency, Elevated sedimentation rate, Tuberculin test reaction abnormal or positive, Anemia and Cushing syndrome.

**Conclusions:**

Data on a large number (> 60,000) of patients with a diagnosis of RA were used to analyze and to list a large number (> 2,000) of co-morbidities. Rank-ordering of RRs of diagnostic codes is a tool to identify quickly many conditions associated with RA.

## Background

In post-marketing surveillance, often questions arise about characteristics of the patient group for which a drug is indicated, such as the age distribution and co-morbidities to be expected [1,2]. Therapeutic risk management involves an understanding of characteristics specific to a group of patients for whom a drug will be prescribed. Often, some safety issues of a new product are predictable; however after market introduction in practice often adverse events are reported that were not foreseen [3,4]. A description of all co-morbidities seen in the target population would help better understand the patient population and thus, provide some context for new safety issues.

Referring to Risk Management Plans (RMPs) submitted to regulatory authorities, a guide on pharmacovigilance published by the Medicines and Healthcare products Regulatory Agency in the UK comments that some RMPs tend to focus on what is already known rather than identifying the areas in which information is lacking [5]. It was also noted that the relevance of the epidemiology of the disease to the target indication is often not sufficiently considered.

To help address these issues, data on large groups from the general population are needed. Increasingly in the past years, health-care databases have become available that enable the study of large numbers of patients with follow-up data. The US Food and Drug Administration Amendment Act of 2007 (FDAAA) called for the establishment, under the auspices of an independent foundation, of a set of databases of health insurance claims data for 100 million people by 2012 [6,7]. Thus, data on sufficient numbers of patients are becoming more available to better understand the co-morbidities in groups or subgroups of patients with a target disease.

Listing co-morbidities, rank-ordered by prevalence, will show various conditions associated with the disease of interest; these associations may be either known, or not yet established. Additionally, such data on a patient group will reflect the target group for a product. The target indication is often restricted by criteria relating to severity of disease or by treatments given earlier. While healthcare databases usually do not have sufficient data on stage or severity of a specific disease, prescribed medication is usually well covered. A subgroup within a healthcare database can be selected, identified by disease codes and restricted by criteria on medication. Information gathered on age/sex profile, co-morbidities and co-medication of such a group should help to describe disease epidemiology specific for a target indication.

Rheumatoid arthritis is a chronic condition characterized by inflammation and modulation of immunity, affecting many organs apart from the loco-motor system. Various studies have described associations of RA with selected morbidities such as infectious conditions [8-10] and cardiovascular disease [11-14]. Because of this potential association with many conditions, it was chosen as the target condition to describe unselected co-morbidity in a data-driven way. The objective was to quantify a large number of co-morbidities associated with RA, as compared to a control group. Associations found should not be assumed to be causal; however they can provide a base for further analyses and for hypothesis testing in different data sets.

## Methods

Thomson's MarketScan Commercial Claims and Encounters Research Database and the Medicare Supplemental from January 1, 1999 through June 30, 2007 was the data source for this analysis. Marketscan is composed of claims submitted to health plans which have contracts with large private employers or with public organizations in the United States. The longitudinal database covers, at the patient level, all inpatient, outpatient, and prescription claims, as long as employees stay enrolled. It consists of employer- and health plan sourced data. Nearly 18 million individuals are included in the 2006 database: employees, their spouses, and dependents. Healthcare for these individuals is provided under a variety of fee-for-service, fully capitated (i.e. set amount per person), and partially capitated health plans. Medical claims are linked to outpatient prescription drug claims and person-level enrollment information. This is one of the largest collections of patient data in the US with over four billion patient records. It includes 77 contributing employers and 12 contributing health plans, with 126 unique carriers, and Medicaid data from eight states [15]; Medicaid covers individuals and families with low income and resources. Elderly are well represented, through the inclusion of groups covered by Medicare, the US social insurance program for people who are aged 65 years and over.

MarketScan research databases meet or exceed requirements of the US Health Insurance Portability and Accountability Act (HIPAA) of 1996. The MarketScan databases underwent a statistical analysis by a third party confirming that HIPAA requirements for fully de-identified data sets were met. Thus, data use met HIPAA criteria for anonymous and aggregate research analysis and reporting of data derived from clinical records not requiring specific patient consent or ethical approval.

Cases were defined as persons with at least two claims for RA [codes ICD 9: 714.0*, 714.1*, 714.2*, and 714.3*.] that were non-diagnostic (i.e. not blood, lab, radiological claims) with active insurance status on June 30, 2007. At least one diagnosis of RA had to be before July 1 2006 and the index date was defined as the earliest claim containing the diagnosis. The control group was patients who had at least two claims for eczema/dermatitis [codes ICD 9: 690.*, 691.*,692.*] with the same criteria as the cases for insurance status and timing of the claims. Controls were matched 1:1 to the cases, by age, gender and health plan [Medicare or not] using propensity scores.

Propensity matching was done using a logistic regression model on dependent variable RA = 0,1 where 1 = in RA cohort and with explanatory variables: age, sex, and health plan (Medicare or not). The propensity score is the probability of being in the RA cohort. Then, one control was selected for each case based on a best match algorithm, using 8 digits of the probability value, then 7 digits, etc. [16] Each control was used only once. SAS version 8.2 was used for all analyses.

All co-morbidities with a level 5 - i.e. the most detailed - ICD9 (diagnostic) code were identified in the case and control groups in the one-year window from July 1, 2006 through June 30, 2007. A one-year period prevalence was calculated for each co-morbidity for both case and control groups. Occurrence of each co-morbidity was counted only once for each patient.

Also, a set of relative risks (RR) was calculated for patients aged at least 16 years old as of RA index date, by dividing the one-year period prevalence of each co-morbidity in the RA group with the corresponding prevalence in the controls. Thus, the RR represents here a ratio of one-year prevalences. After this, the various co-morbidities were rank-ordered by magnitude of RR; 95% confidence intervals were added.

ICD9 codes covering arthropathies and related disorders [710-719], dorsopathies [720-724] and rheumatism, excluding the back [725-729] were excluded from the analysis (as likely related/part RA), also mechanical complications of internal orthopedic device, implant and graft [996.4]. In order to obtain stable estimates, rank-ordering was done for non-rare diagnoses (occurring in at least 20 persons in control group).

## Results

Records for 62,681 persons with RA were selected (all ages). Mean age was 59.0 Y, with 73.8% female, 35% Medicare-covered. A total of 6,897 different ICD9 diagnostic codes were recorded in this group, of which 2,220 occurred in 20 or more persons with RA.

The most prevalent co-morbidities in the cohort with RA are listed in Table 1. At the more detailed level of the ICD9 classification (level 5), the most commonly registered co-morbidities were hypertension not otherwise specified (NOS) (20.4%), benign hypertension (19.9%), chest pain NOS (13.9%), and hyperlipidemia not elsewhere classified (NEC)/NOS (13.9%).

**Table 1:** Most common co-morbidities in cohort with rheumatoid arthritis (N = 62,681, all ages)*

Condition (ICD-9 code)	Number of persons	Percent
4019 Hypertension NOS	12,773	20.4%
4011 Benign hypertension	12,482	19.9%
78650 Chest pain NOS	8,742	13.9%
2724 Hyperlipidemia NEC/NOS	8,690	13.9%
25000 Diabetes Mellitus II without complication	7,504	12.0%
78079 Malaise & fatigue NEC	6,046	9.6%
5990 Urinary tract infection NOS	5,965	9.5%
78900 Abdominal pain unspecified site	5,712	9.1%
7862 Cough	5,649	9.0%
2449 Hypothyroidism NOS	5,524	8.8%
78605 Shortness of breath	5,516	8.8%
4660 Acute bronchitis	5,400	8.6%
2720 Pure hypercholesterolemia	5,046	8.1%
4619 Acute sinusitis NOS	4,833	7.7%
2859 Anemia NOS	4,632	7.4%
73300 Osteoporosis NOS	4,529	7.2%
41401 Coronary atherosclerosis native vessel	4,325	6.9%
53081 Esophageal reflux	4,288	6.8%
7020 Actinic keratosis	4,249	6.8%
73390 Bone & cartilage disease NOS	4,113	6.6%
4659 Acute upper respiratory infection NOS	4,112	6.6%
496 Chronic airway obstruct NEC	4,041	6.4%
36616 Senile nuclear cataract	3,914	6.2%
78609 Respiratory abnormality NEC	3,605	5.8%
7840 Headache	3,246	5.2%
41400 Coronary atherosclerosis unspec. vessel	3,002	4.8%

* Excluded arthritis/RA-related codes ICD 710-729; 996.4.

NOS: Not otherwise specified NEC: Not elsewhere classified

**Figure 1:**
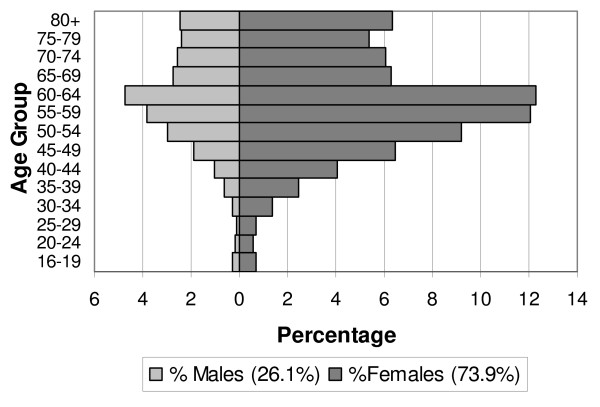
Age/sex distribution of rheumatoid arthritis cases 16 years and older (N = 61,591)

**Figure 2:**
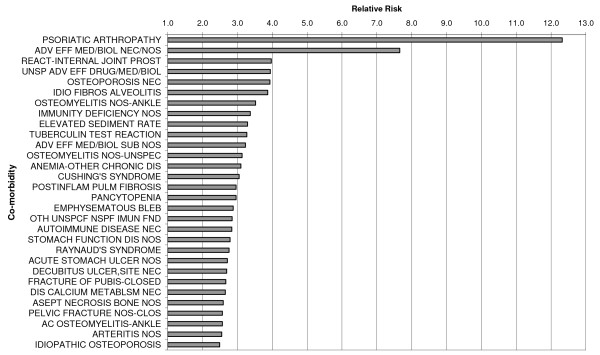
Top 30 Relative risks of co-morbidities in patients with rheumatoid arthritis 16 years and older as compared with control group (N = 61,591 for each group)

A total of 61,591 persons with RA were aged at least 16 years and subsequently matched with the control group. Demographic distribution for RA cases is shown in Figure 1. The demographic distribution of the controls was identical. Co-morbidities strongly associated with RA in the case group compared to controls are shown in Table 2 and Figure 2. A full set of co-morbidities is provided in additional File 1: Rank-ordered relative risks of co-morbidities in patients with rheumatoid arthritis.

**Table 2:** Top 30 Relative risks of co-morbidities in patients with rheumatoid arthritis 16 years and older as compared with control group (N = 61,591 for each group)

ICD9	Co-morbidity Condition	Persons with RA (N)	Percent	RR	95% CI
6960	Psoriatic arthropathy	1,540	2.5%	**12.3**	10.5-15.1
99529	Adverse effect medicinal and biologic substance NEC/NOS	360	0.6%	**7.7**	5.7-10.4
99666	Reaction-internal joint prosthesis	131	0.2%	**4.0**	2.7-5.8
9952	Unspecified adverse effect drug, medicinal and biol substance properly administered	717	1.2%	**3.9**	3.4-4.7
73309	Osteoporosis NEC	479	0.8%	**3.9**	3.2-4.8
5163	Idiopathic fibrosing alveolitis	205	0.3%	**3.9**	2.9-5.2
73027	Osteomyelitis NOS-ankle	148	0.2%	**3.5**	2.5-5.0
2793	Immunity deficiency NOS	81	0.1%	**3.4**	2.1-5.3
7901	Elevated sediment rate	171	0.3%	**3.3**	2.4-4.5
7955	Tuberculin test reaction abnormal or positive	239	0.4%	**3.3**	2.5-4.3
99520	Adverse effect medicinal and biologic substance NOS	708	1.1%	**3.2**	2.8-3.8
73020	Osteomyelitis NOS-unspec	119	0.2%	**3.1**	2.2-4.5
28529	Anemia-of other chronic illness	415	0.7%	**3.1**	2.6-3.8
2550	Cushing's syndrome	61	0.1%	**3.0**	1.8-5.1
515	Postinflammatory pulmonary fibrosis	1,221	2.0%	**3.0**	2.7-3.4
2841	Pancytopenia	80	0.1%	**3.0**	1.9-4.6
4920	Emphysematous BLEB	72	0.1%	**2.9**	1.8-4.5
79579	Other and unspecified nonspecific immunologic findings	373	0.6%	**2.8**	2.3-3.5
2794	Autoimmune disease NEC	142	0.2%	**2.8**	2.1-3.9
5369	Stomach function disorder NOS	67	0.1%	**2.8**	1.8-4.4
4430	Raynaud's syndrome	251	0.4%	**2.8**	2.2-3.5
53130	Acute stomach ulcer NOS	76	0.1%	**2.7**	1.8-4.2
70709	Decubitus ulcer, site NEC	97	0.2%	**2.7**	1.8-4.0
8082	Fracture of pubis-closed	136	0.2%	**2.7**	1.9-3.7
27549	Disorders of calcium metabolism NEC	69	0.1%	**2.6**	1.7-4.2
73340	Aseptic necrosis bone NOS	75	0.1%	**2.6**	1.7-4.0
8088	Pelvic fracture NOS-closed	121	0.2%	**2.6**	1.8-3.6
73007	Acute osteomyelitis-ankle	77	0.1%	**2.6**	1.7-3.9
4476	Arteritis NOS	247	0.4%	**2.5**	2.0-3.2
73302	Idiopathic osteoporosis	182	0.3%	**2.5**	1.9-3.3

* Aged at least 16 years, matched controls with Dermatitis/Eczema diagnosis.

Only includes conditions for which there were at least 20 controls Excluded Arthritis/RA-related codes ICD 710-729;996.4.

NOS: Not otherwise specified NEC: Not elsewhere classified

Additional file 2 shows top 30 of relative risks as well as odds ratios of co-morbidities in patients with RA; comparator group had dermatitis/eczema diagnosis

Additional file 3 shows same analysis as additional file 2, however with a different (random) comparator group.

Strong associations with RA (RR > 3) were found for psoriatic arthropathy, adverse effect to medicine/biological, reaction to internal joint prosthesis, unspecified adverse effect, osteoporosis, idiopathic fibrosing alveolitis, osteomyelitis, immune deficiency, elevated sedimentation rate, tuberculin test reaction, anemia and Cushing syndrome [Table 2, Figure 2]. As described in the Methods section, a set of ICD codes covering conditions of the loco-motor system was excluded from the analysis. All RRs in Table 2 were statistically significant. The full set of co-morbidities as shown in additional File 1 includes many with a RR < 1, i.e. associated with the control group. Many of these are skin conditions.

## Discussion

Data on a large number of patients (> 60,000) with a diagnosis of rheumatoid arthritis were available, with a large number (> 6,000) of different ICD9 diagnostic codes. Most prevalent co-morbidities were in the cardiovascular area: hypertension, chest pain and hyperlipidemia. The rank-ordering of relative risks shows a variety of conditions to be associated with RA. Some of these have been reported previously such as fibrosing alveolitis [17,18] and acute stomach ulcer [19,20]; it also shows a strong and statistically significant association with osteomyelitis. Published studies on even relatively large cohorts of RA patients typically show only a few cases of this rare but severe infection. [21-23]. Also, of note, is the strong association found with 'adverse effect to medication'; this is an ICD-9 category with no information on the nature of the reaction or the product imputed. RR for osteoporosis is markedly increased, also for various fractures; this is in line with the findings of Van Staa et al based on the General Practice Research Database in the UK [24]. Osteoporosis is likely a direct consequence of RA, decreased physical activity and treatments with corticosteroids [25]. The association of RA with psoriatic arthropathy may be a consequence of similarities of signs of these conditions in their early stages where follow-up of patients would lead to a change of diagnosis. Psoriatic arthropathy is in the ICD category of skin conditions; hence it was included in Table 2. The many diagnostic codes that are part of ICD categories that cover arthritis and other conditions of the locomotor system have been excluded as these were not the focus of the co-morbidity analysis. However these are included in additional File 1: Rank-ordered relative risks of co-morbidities in patients with rheumatoid arthritis.

All RRs shown in Table 2 are statistically significant with lower boundaries of the 95% confidence interval above 1. This is a result of conditions being both strongly associated with RA and not too rare (at least 20 cases in control group).

It should be stressed that some less common events may be highly relevant from a drug safety perspective. A data set covering information on patients with RA or any other condition may indeed be used for analysis of any event of interest, irrespective of its rate of occurrence.

The control group included a group of patients with a non-systemic chronic condition (eczema/dermatitis) occurring in a broad age range, but with no direct relation to RA.

The choice of a control group of patients with another chronic condition should help to control for bias in detecting co-morbidities as both groups have exposure to health care. It should be noted however that some diagnoses that are relatively common both in the general population and in the RA population such as various skin conditions have a RR under 1 due to an association with the control group. For example 'pruritic disorders NOS' had a RR of 0.40.

Various control groups may be selected, depending on the types of outcomes of interest. For example, some diagnoses are typically made in the setting of out-patient clinics, other ones during hospital stays. The choice of control groups should take these differences into account.

As with any data source, claims data have limitations which are due both to the nature of administrative claims for payment purposes and convenience samples as opposed to random samples of the population. As such, they may contain biases or fail to generalize well to other populations. Clinical knowledge about comorbidities of RA or eczema/dermatitis is likely to drive the conditions that clinicians test for and deem worth recording. Information on important aspects such as smoking behaviour and ethnicity is missing. Associations found should not be considered to be causal. Rank-ordering of RRs of diagnostic codes is a tool to quickly identify characteristics specific to patients with a condition of interest. This can be applied to various subgroups of patients with the condition selected such as patients treated with different pharmaceutical products of interest. Comparisons of such groups can show whether products are prescribed to patients with different co-morbidity patterns prior to treatment. Understanding such selective prescribing can help in the interpretation of reported adverse effects of new products, as a part of good risk management practice.

## Conclusions

Rank-ordering of relative risks of diagnostic codes is a tool to quickly identify co-morbidities specific to a patient group of interest, in this case patients with rheumatoid arthritis. Conditions with a strong (RR > 3), statistically significant association with RA included osteomyelitis; the large cohort of RA patients (> 60,000) covered makes it possible to quantify risk for this and other uncommon co-morbiditities.

## Competing interests

The three authors are employees of Roche. Roche has products for rheumatoid arthritis, the disease described in this publication.

## Authors' contributions

HP and NJR conceived the study. HP drafted the manuscript. DM carried out the analyses and helped to draft the manuscript. All authors read and approved the final manuscript.

## Pre-publication history

The pre-publication history for this paper can be accessed here:

http://www.biomedcentral.com/1471-2474/11/247/prepub

## Supplementary Material

Additional file 1 **Rank-ordered Relative Risks of co-morbidities in patients with rheumatoid arthritis. RR as compared with control group (N = 61,591 for each group). 16 years and older. Arthritis/RA-related codes included**. Conditions included for which there were at least 20 controls. Number of RA cases in table may be less than 20 if RR < 1, i.e. if fewer cases than controls have condition. Shown: ICD9 codes, description of co-morbidity, number of persons in group with RA, percentage of group with RA, Relative Risk. NOS: Not otherwise specified NEC: Not elsewhere classified Click here for file

Additional file 2 **Top 30 Relative risks and Odds ratios in patients with rheumatoid arthritis. Comparator group with Dermatitis/Eczema diagnosis (N = 61,591 for each group). 16 years and older. RR + OR with 95% confidence intervals**. Conditions included for which there were at least 20 controls. Shown: ICD9 codes, description of co-morbidity, numbers of persons with co-morbidity in RA and control groups, Relative Risk, numbers of persons without co-morbidity in RA and control groups, Odds Ratio. 95% Confidence Intervals Click here for file

Additional file 3 **Top 30 Relative risks and Odds ratios in patients with rheumatoid arthritis. Comparator random group (N = 61,591 for each group). 16 years and older RR + OR with 95% confidence intervals**. Conditions included for which there were at least 20 controls. Shown: ICD9 codes, description of co-morbidity, numbers of persons with co-morbidity in RA and control groups, Relative Risk, numbers of persons without co-morbidity in RA and control groups, Odds Ratio. 95% Confidence Intervals Click here for file
